# Associations Between Factors Across Life and One-Legged Balance Performance in Mid and Later Life: Evidence From a British Birth Cohort Study

**DOI:** 10.3389/fspor.2020.00028

**Published:** 2020-04-07

**Authors:** Joanna M. Blodgett, Rachel Cooper, Daniel H. J. Davis, Diana Kuh, Rebecca Hardy

**Affiliations:** ^1^MRC Unit for Lifelong Health and Ageing at UCL, London, United Kingdom; ^2^Department of Sport and Exercise Sciences, Musculoskeletal Science and Sports Medicine Research Centre, Manchester Metropolitan University, Manchester, United Kingdom; ^3^CLOSER, Institute of Education, UCL, London, United Kingdom

**Keywords:** balance, aging, life course, risk factor, epidemiology

## Abstract

**Introduction:** Despite its associations with falls, disability, and mortality, balance is an under-recognized and frequently overlooked aspect of aging. Studies investigating associations between factors across life and balance are limited. Understanding the factors related to balance performance could help identify protective factors and appropriate interventions across the life course. This study aimed to: (i) identify socioeconomic, anthropometric, behavioral, health, and cognitive factors that are associated with one-legged balance performance; and (ii) explore how these associations change with age.

**Methods:** Data came from 3,111 members of the MRC National Survey of Health and Development, a British birth cohort study. Multilevel models examined how one-legged standing balance times (assessed at ages 53, 60–64, and 69) were associated with 15 factors across life: sex, maternal education (4 years), paternal occupation (4 years), own education (26 years), own occupation (53 years), and contemporaneous measures (53, 60–64, 69 years) of height, BMI, physical activity, smoking, diabetes, respiratory symptoms, cardiovascular events, knee pain, depression and verbal memory. Age and sex interactions with each variable were assessed.

**Results:** Men had 18.8% (95%CI: 13.6, 23.9) longer balance times than women at age 53, although this difference decreased with age (11.8% at age 60–64 and 7.6% at age 69). Disadvantaged socioeconomic position in childhood and adulthood, low educational attainment, less healthy behaviors, poor health status, lower cognition, higher body mass index (BMI), and shorter height were associated with poorer balance at all three ages. For example, at age 53, those from the lowest paternal occupational classes had 29.6% (22.2, 38.8) worse balance than those from the highest classes. Associations of balance with socioeconomic indicators, cognition and physical activity became smaller with age, while associations with knee pain and depression became larger. There were no sex differences in these associations. In a combined model, the majority of factors remained associated with balance.

**Discussion:** This study identified numerous risk factors across life that are associated with one-legged balance performance and highlighted diverse patterns of association with age, suggesting that there are opportunities to intervene in early, mid and later life. A multifactorial approach to intervention, at both societal and individual levels, may have more benefit than focusing on a single risk factor.

## Introduction

From getting out of bed in the morning to sitting, standing and walking throughout the day, balance is a crucial component of everyday life (Muir et al., 2010). Poor balance is linked with several adverse health outcomes, perhaps most notably increased falls risk (Ganz et al., 2007), but also with increased risk of disability, fractures, hospitalization, and premature mortality (Cooper et al., 2011b, 2014; Nofuji et al., 2016; Keevil et al., 2018). Despite the growing awareness of the importance of balance in aging—as reflected in recent physical activity guidelines (Centre for Ageing Better, 2018; US Department of Health Human Services., 2018; Department of Health Social Care., 2019)—the life course epidemiology of balance performance has been under-investigated compared with other measures of physical capability such as grip strength and chair rise performance.

In the few studies that have examined factors across life in relation to balance performance, several associations have been found. Across a range of ages, males tend to have better balance performance than females (Wolfson et al., 1994; Schultz et al., 1997; Cooper et al., 2011a; Kim et al., 2012). Low socioeconomic position (SEP) has been found to have a negative cumulative association with balance performance, with an additive effect of low SEP in childhood and adulthood on risk for poor balance in later life (Birnie et al., 2011b; Strand et al., 2011a). Smoking history (Strand et al., 2011b), low cognitive ability in both childhood and adulthood (Kuh et al., 2009a; Blodgett et al., 2020), higher levels of depression (Nitz et al., 2005), and low levels of physical activity (Cooper et al., 2011c, 2015; Chang et al., 2013), have also been shown to be associated with poor balance.

These previous studies have primarily examined associations between a single risk factor and balance ability at one time point. With the exception of our recent study of the association between childhood cognitive ability and balance performance (Blodgett et al., 2020), to our knowledge, no study has examined whether associations change with age. This is a limitation, given that balance is a complex process that relies on sensory input including visual cues, proprioception, vestibular processes as well as muscular strength and cognitive processing (Merla and Spaulding, 1997), and so may be affected by age-related changes, such as increased levels of morbidity and decline in cognitive functioning. In addition, few studies have investigated sex differences in the associations between risk factors and balance ability. This is despite the fact that investigating sex differences in the relationships between different risk factors and balance may help elucidate why men have better average balance performance than women, as the reasons for this are still not fully understood (Maki et al., 1990; Wolfson et al., 1994; Hageman et al., 1995; Schultz et al., 1997; Bryant et al., 2005).

Using a British birth cohort study, previously used to study factors associated with balance at a single age (Kuh et al., 2006, 2009a; Birnie et al., 2011a; Cooper et al., 2011a,c, 2015; Strand et al., 2011a,b; Mulla et al., 2013; Murray et al., 2013; Blodgett et al., 2020), we aimed to investigate associations of socioeconomic, behavioral, health and cognitive risk factors across life with one-legged balance performance over 16 years and assess if these associations change with age or sex. We hypothesized that positive factors such as high SEP, low BMI, participation in healthy behaviors, absence of poor physical and mental health as well as higher adult cognitive ability would be associated with better balance performance. As physical and mental comorbidities become more common with age, we hypothesized that the associations of health status with balance performance would get stronger with age. Conversely, as health status becomes more important, the relative contributions of SEP were hypothesized to decrease.

## Methods

### Sample

The MRC National Survey of Health and Development (NSHD) is an ongoing study of 5,362 individuals born in England, Scotland, or Wales within 1 week in March 1946. Since 1946, study members have been followed up to 24 times in infancy, across childhood, adolescence, and adulthood, most recently at ages 53 (*n* = 2,988), 60–64 (*n* = 2,229), and 69 (*n* = 2,149) using a combination of questionnaires, interviews, and clinical examinations (Kuh et al., 2016). Details of loss to follow-up (e.g., death, emigration, refusal, incapacity) in this sample have been previously described (Blodgett et al., 2020). Ethical approval for the most recent data collection wave (2015) was obtained from Queen Square Research Ethics Committee (14/ LO/1073) and Scotland A Research Ethics Committee (14/SS/1009).

### Assessment of Balance Ability

*One-legged balance performance* was assessed by trained nurses during clinical assessments at ages 53, 60–64, and 69 using standardized protocols. Study members were asked to fold their arms and stand on their preferred leg with their eyes closed for as long as possible up to a maximum of 30 s. If individuals were unable to perform the test, the reason was recorded. In these analyses, individuals who could not perform the test due to health reasons and those who attempted but could not maintain the balance position were given a score of zero. The final analytical sample consisted of individuals with a balance time at one or more ages (*n* = 3,111). The one-legged balance test is considered to be a reliable and valid measure of static balance and has been shown to have high inter-rater and test-retest reliability (Giorgetti et al., 1998; Bohannon, 2006; Springer et al., 2007; Michikawa et al., 2009; Choi et al., 2014; Ortega-Pérez de Villar et al., 2018). Many studies have consistently demonstrated associations between poor one-legged balance performance and higher risk of falls, disability, poor gait speed, frailty and premature mortality (Drusini et al., 2002; Michikawa et al., 2009; Cooper et al., 2010, 2014; Delbaere et al., 2010; Oliveira et al., 2018).

### Assessment of Risk Factors

We selected a set of risk factors *a priori* that had previously been shown to be associated with balance or other measures of physical capability at a single time point in NSHD and other studies (Kuh et al., 2006, 2009a; Birnie et al., 2011b; Cooper et al., 2011a,c, 2015; Strand et al., 2011a,b; Welmer et al., 2012; D'Andréa Greve et al., 2013; Amemiya et al., 2019; Thomas et al., 2019).

#### Socioeconomic Indicators

*Paternal occupational class* (at age 4) and *own occupational class* (reported at age 53 years) were grouped into three categories as distinguished by the Registrar General's Social Classification (Galobardes et al., 2006): (1) I Professional and II Intermediate; (2) III Skilled (non-manual) and III Skilled (manual); and (3) IV Partly skilled and V Unskilled manual. *Maternal education* was classified into four categories: (1) Primary only; (2) Primary and further education; (3) Secondary only; (4) Secondary and further education. Participants reported their highest level of *educational attainment* by age 26, which was categorized as degree or higher, advanced secondary qualifications generally attained at 18 years (GCE A level or Burnham B), ordinary secondary qualifications generally attained at 16 years, (e.g., GCE O level or Burnham C), below ordinary secondary qualifications, or none.

#### Anthropometric Indicators (Ages 53, 60–64, 69)

*Height (m)* and *BMI (kg/m*^2^*)*, derived from height and weight measurements ascertained by nurses using standardized protocols, were used (Braddon et al., 1986).

#### Behavioral Risk Factors (Ages 53, 60–64, 69)

Individuals self-reported their *leisure time physical activity participation* (never, 1–4 times/month, 5+ times/month) and their *smoking status* (never, past smoker, current smoker) (Kuh et al., 2009b; Strand et al., 2011b). Current and past smokers were defined as those who smoked at least one cigarette a day for 12 months or more.

#### Health Status (Ages 53, 60–64, 69)

Current health conditions (yes/no for each) were ascertained using a series of self-reported questions on *history of diabetes, cardiovascular events, respiratory symptoms, and knee pain* (Kuh et al., 2005; Cooper et al., 2014). *Symptoms of depression and anxiety* were assessed using the 28-item self-reported General Health Questionnaire; each item was scored from 1 to 4 and summed together (range: 0–84) (Goldberg and Hillier, 1979).

#### Cognitive Ability (Ages 53, 60–64, 69)

Verbal memory was assessed using a 15-item word list. Each word was presented for 2 s before individuals were instructed to write down as many words as they could remember. This was repeated over three identical trials and the number of words recalled during each trial were summed (range: 0–45). To minimize any practice effects, two word lists were rotated between follow-up assessments (Davis et al., 2017).

### Statistical Analyses

Sex differences in each risk factor were assessed using *t*-tests or chi-square tests, as appropriate, and described by the mean (±SD) or proportion (*n*). Separate multilevel models were used to examine the associations between each risk factor (independent variable) and log transformed balance time (dependent variable) in the maximal available sample size. Cross-sectional associations were assessed for time-varying covariates (e.g., anthropometric, behavioral, health, cognitive factors), whereas SEP measures were based on reports from one age. Balance times at each age (level 1) were nested within individuals (level 2) and both the intercept and slope were modeled as random effects. As the sample was age-homogenous, age was employed as a linear time metric and was centered at age 53 (intercept); age 63 was utilized as the time integer for age 60–64 (Kuh et al., 2019; Blodgett et al., 2020). Balance times were log-transformed due to the skewed distribution of balance. Non-linearity of the association between each risk factor and balance was assessed using likelihood ratio tests.

A variable-by-sex interaction term was estimated, with subsequent models stratified by sex if there was evidence of an interaction. An interaction between age and each risk factor was added to the model to test whether the association between each risk factor and balance changes with age. Age interactions were considered if *p* < 0.05; an alpha of 0.05 was used for both age and sex interactions in order to parsimoniously build each model. Finally, all risk factors and significant interaction terms were included in a combined model. To account for the non-random events of mortality and attrition (not due to death), the model was adjusted for separate binary indicators of both death and attrition (not due to death) between ages 53 and 69. This approach minimizes the correlation between non-random loss to follow up and poorer performance on the balance test, thus reducing bias in the other estimates (Botoseneanu and Liang, 2012; Botoseneanu et al., 2013). All estimates are presented as sympercents (i.e., as % change) to aid interpretation (Cole and Kryakin, 2000). Stata 14 was used for all statistical analyses.

## Results

Characteristics of the sample are described in Table 1. Men were taller than women, had higher adult SEP, higher educational attainment, lower verbal memory, and were more likely to have a history of smoking. Men also had a higher prevalence of diabetes and CVD events, although women reported higher prevalence of knee pain and symptoms of anxiety and depression.

**Table 1:** Characteristics of analytical sample (*n* = 3,111), MRC National Survey of Health and Development.

		**Men (*n* = 1,550)**	**Women (*n* = 1,561)**	**Tests of sex differences (*p*-value)**
**ONE-LEGGED BALANCE TIME (s), MEDIAN (Q1, Q3)**, ***n***				
Age 53		5 (3, 10), *n* = 1421	4 (3, 7), *n* = 1,476	<0.001
Age 60-64		3.57 (2.35, 5.53), *n* =1,055	3.16 (2.16, 4.72), *n* = 1,148	<0.001
Age 69		2.94 (1.84, 4.78), *n* = 1,037	2.72 (1.69, 4.15), *n* = 1,079	<0.005
**SOCIOECONOMIC INDICATORS**, ***n*** **(%)**				
**Paternal occupational class**				
I Professional/II Intermediate		407 (27.6)	383 (26.0)	0.56
III Skilled (non-manual or manual)		692 (46.9)	716 (48.7)	
IV Partly skilled/V Unskilled		377 (25.5)	372 (25.3)	
**Maternal education**				
Secondary and further education		162 (11.74)	169 (12.2)	0.49
Secondary only		167 (12.1)	153 (11.0)	
Primary and further education		213 (15.4)	193 (13.90)	
Primary only		838 (60.7)	873 (62.9)	
**Highest household occupational class**				
I Professional/II Intermediate		788 (51.6)	559 (36.1)	<0.001
III Skilled (non-manual or manual)		578 (37.8)	659 (42.6)	
IV Partly skilled/V Unskilled		162 (10.6)	329 (21.3)	
**Educational attainment at age 26**				
Degree or higher		212 (14.5)	81 (5.5)	<0.001
GCE A level or Burnham B		408 (27.9)	343 (23.3)	
GCE O level or Burnham C		211 (14.4)	377 (25.6)	
Sub GCE		92 (6.3)	134 (9.1)	
None attempted		540 (36.9)	537 (36.5)	
**ANTHROPOMETRY, MEAN (SD)**				
**Height (m)**				
Age 53		1.75 (0.07), *n* = 1,436	1.62 (0.06), *n* = 1,498	<0.001
Age 60–64		1.75 (0.09), n=1062	1.62 (0.06), n=1159	<0.001
Age 69		1.73 (0.09), *n* = 1,023	1.61 (0.06), *n* = 1,077	<0.001
**BMI (kg/m**^**2**^**)**				
Age 53		27.4 (4.0), *n* = 1,435	27.4 (5.5), *n* = 1,486	0.89
Age 60–64		27.9 (4.1), *n* = 1,061	27.9 (5.5), *n* = 1,158	0.92
Age 69		28.2 (4.6), *n* = 1,040	28.2 (5.7), *n* = 1,081	0.91
**BEHAVIORAL RISK FACTORS**, ***n*** **(%)**				
**Leisure time physical activity**				
Age 53	None	693 (47.9)	761 (50.4)	0.18
	1–4 times/month	270 (18.7)	245 (16.2)	
	5+ times/month	485 (33.5)	503 (33.3)	
Age 60–64	None	681 (65.2)	716 (62.9)	0.52
	1–4 times/month	137 (13.1)	162 (14.2)	
	5+ times/month	227 (21.7)	261 (22.9)	
Age 69	None	711 (59.9)	777 (61.33)	0.08
	1–4 times/month	135 (11.4)	170 (13.42)	
	5+ times/month	341 (28.7)	320 (25.3)	
**Smoking status**				
Age 53	Current	343 (23.6)	339 (22.5)	<0.001
	Previous smoker	737 (50.8)	671 (44.5)	
	Never smoker	371 (25.6)	499 (33.1)	
Age 60–64	Current	137 (12.3)	142 (11.8)	<0.001
	Previous smoker	663 (59.5)	629 (52.1)	
	Never smoker	314 (28.2)	436 (36.1)	
Age 69	Current	123 (10.3)	111 (8.8)	<0.001
	Previous smoker	756 (63.5)	723 (57.2)	
	Never smoker	311 (26.1)	430 (34.0)	
**HEALTH STATUS**, ***n*** **(%)**				
Diabetes	Age53	57 (3.1)	43 (2.4)	0.18
	Age60–64	129 (10.1)	99 (7.2)	<0.01
	Age69	175 (13.7)	136 (10.0)	<0.005
CVD events	Age53	85 (5.8)	48 (3.2)	<0.01
	Age60–64	131 (11.5)	62 (5.1)	<0.001
	Age69	193 (17.6)	114 (10.0)	<0.001
Respiratory symptoms	Age 53	292 (19.9)	276 (18.2)	0.22
	Age 60–64	233 (20.1)	224 (18.2)	0.15
	Age 69	264 (24.5)	266 (22.4)	0.23
Knee pain	Age 53	226 (15.5)	310 (20.6)	<0.001
	Age 60–64	216 (20.3)	288 (24.6)	0.01
	Age 69	190 (18.1)	241 (22.1)	0.02
Depression/anxiety	Age 53	15.2 (7.3), *n* = 1,051	17.8 (8.9), *n* = 1,137	<0.001
	Age 60–64	15.7 (8.6), *n* = 1,407	18.9 (10.3), *n* = 1,470	<0.001
	Age 69	14.1 (7.5), *n* = 1025	16.2 (8.2), *n* = 1068	<0.001
**VERBAL MEMORY SCORES, MEAN (SD)**				
	Age 53	23.0 (6.2), *n* = 1,397	24.9 (6.2), *n* = 1473	<0.001
	Age 60–64	23.0 (5.9), *n* = 1,023	25.4 (6.1), *n* = 1,127	<0.001
	Age 69	21.2 (6.0), *n* = 1,005	23.1 (6.0), *n* = 1057	<0.001

### Sex, Age, and Balance

**Figure 1:**
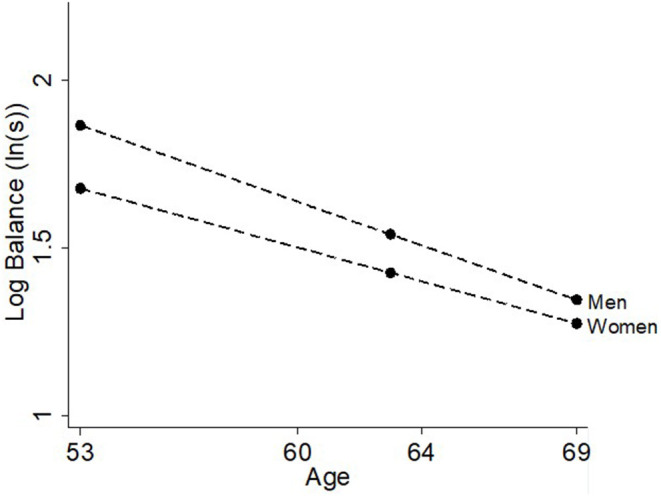
Differences in balance time at ages 53, 60–64, and 69 years by sex.

Women had 18.8% (95%CI: 13.6, 23.9%; Table 2, Figure 1) worse balance performance than men at age 53. The interaction between age and sex indicated that for every additional year increase in age, the sex difference in balance decreased by 0.7% (0.3, 1.2%). Thus, at ages 63 and 69, respectively, women had 11.4% (7.6, 15.2%) and 7.0% (2.1, 11.9%) lower balance times than men. Despite the sex differences in balance performance across time, there were no interactions between sex and any of the risk factors investigated.

**Table 2:** Associations between risk factors and balance performance in multilevel models.

		**Mean difference in % balance time at age 53 (intercept)**		Age (years)*risk factor interaction	
**Risk factors^a^**	***n* participants (*n* observations)**	**Coefficient (%) (95% CI)**	***p-value***	**Coefficient (%) (95% CI)**	***p-value***
1: Sex [female vs. male (ref)]	3,111 (obs = 7,216)	−18.8 (−23.9, −13.6)	<0.001	0.7 (0.3, 1.2)	<0.001
2: Paternal occupational class^b^	2,947 (obs = 6,838)	−14.8 (−18.4, −11.1)	<0.001	0.5 (0.2, 0.8)	<0.001
(per 1 level change)					
3: Maternal education^c^	2,768 (obs = 6,424)	−11.3 (−23.8, −8.8)	<0.001	0.4 (0.2, 0.6)	<0.001
(per 1 level change)					
4: Education at age 26^d^	2,935 (obs = 6,830)	−11.1 (−12.9, −9.3)	<0.001	0.3 (0.2, 0.5)	<0.001
(per 1 level change)					
5: Own occupational class^b^	3,075 (obs = 7,167)	−15.2 (−17.9, −12.6)	<0.001	-	-
(per 1 level change)					
6: Height (cm)					
linear term	3,090 (obs = 7,144)	12.5 (6.4, 18.7)	<0.001	-	-
quadratic term		−0.04 (−0.05, −0.02)	<0.001		
7: BMI (per kg/m^2^)	3,083 (obs = 7,150)	−2.8 (−3.1, −2.5)	<0.001	-	-
8: Leisure time physical activity^e^	3,094 (obs = 6,960)				
1–4 times/week		23.9 (17.3, 30.5)	<0.001	−0.7 (−1.3, 0.02)	0.04
5+ times/week		23.3 (18.0, 28.7)	<0.001	−0.4 (−0.9, 0.1)	0.12
9: Smoking^f^	3,092 (obs = 6,996)	6.1 (3.3, 8.9)	<0.001	-	-
(per 1 level change)					
10: Diabetes^g^	3,111 (obs = 7,214)	18.0 (11.6, 24.4)	<0.001	-	-
11: CVD events^g^	3,072 (obs = 6,895)	19.1 (12.9, 25.4)	<0.001	-	-
12: Respiratory symptoms^g^	3,062 (obs = 6,634)	8.6 (4.5, 12.7)	<0.001	-	-
13: Knee pain ^g^	3,108 (obs = 7,173)	10.8 (4.6, 17.0)	<0.001	−0.7 (−1.3, −0.01)	0.02
14: Symptoms of depression/anxiety^h^	3,071 (obs = 7,032)	5.3 (2.8, 7.7)	<0.001	0.3 (0.04, 0.5)	<0.02
(per 1 SD)					
15: Verbal memory^h^	3,035 (obs = 6,979)	13.4 (11.0, 15.9)	<0.001	−0.4 (−0.6, −0.2)	<0.05
(per 1SD)					

a *all models adjusted for sex as no evidence of sex interactions (see Table 3)*.

b *ref: I Professional or II Intermediate*.

c *ref: Secondary and further education*.

d *ref: Degree or higher*.

e *ref: none in last 4 weeks*.

f *ref: current smoker*.

g *ref: individuals with no health condition*.

h *SD estimates at each age are provided in Table 1*.

### Socioeconomic Indicators and Balance

**Figure 2:**
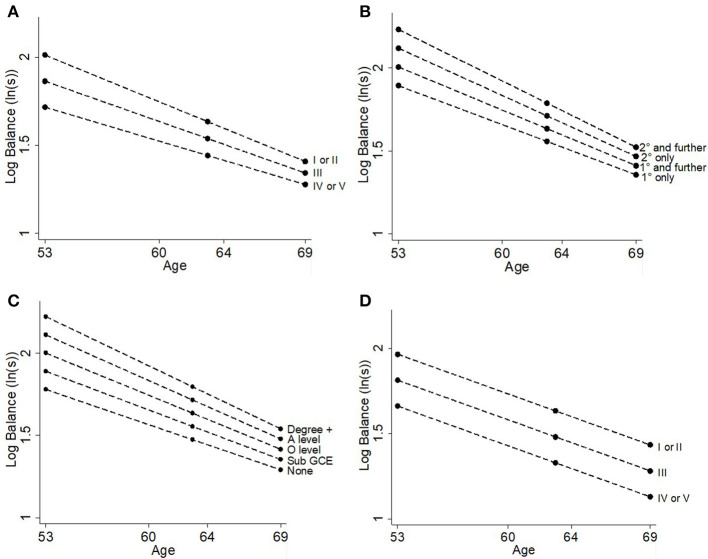
Differences in balance time at ages 53, 60–64, and 69 years by **(A)** paternal occupational class, **(B)** maternal education, **(C)** own education, and **(D)** own occupational class.

The results of the likelihood ratio tests for deviations from linearity suggested that all four socioeconomic indicators could be modeled as continuous variables. More disadvantaged SEP for all four indicators—paternal occupation, maternal education, own education, own occupation—was associated with worse balance times (Table 2, Figure 2). For example, more disadvantaged paternal occupational class was associated with 14.8% (11.1, 18.4%; Table 2, Figure 2A) poorer balance time for each subsequent level. The associations with paternal occupational class, maternal education and own educational attainment all became smaller with age (all *p* < 0.001, Table 2, Figure 2), however there was no interaction between own occupational class and age (*p* = 0.1).

### Anthropometric Indicators and Balance

**Figure 3:**
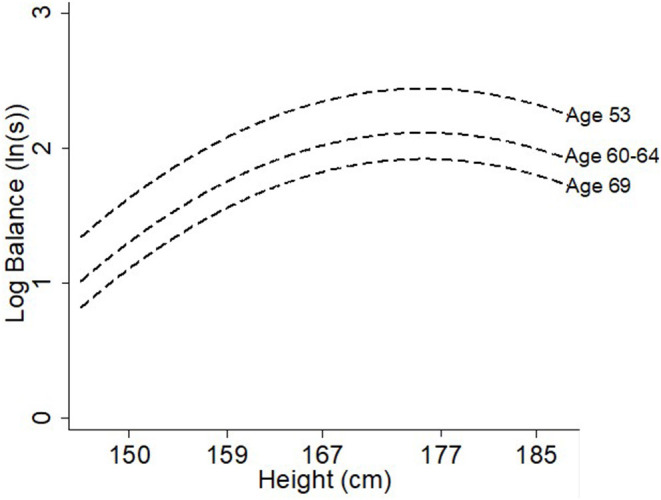
Differences in balance time at ages 53, 60–64, and 69 years by height (cm).

**Figure 4:**
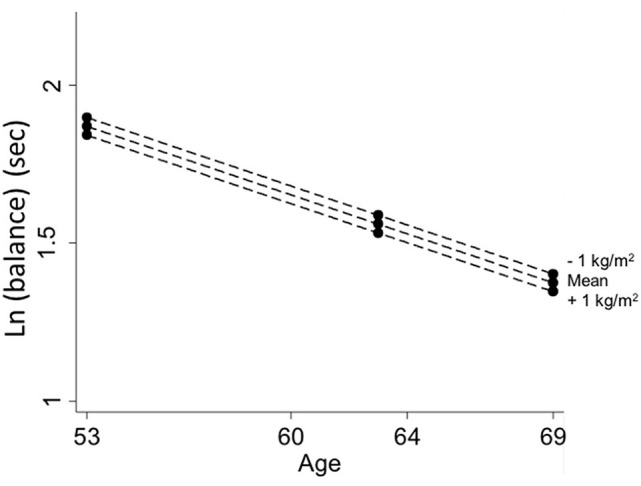
Differences in balance time at ages 53, 60–64, and 69 years by BMI (kg/m^2^).

Height had a quadratic association with balance such that taller individuals had better balance times than shorter individuals although this association plateaued at the tallest heights (see Table 2, Figure 3). BMI had an inverse linear association with balance, where every additional kg/m^2^ was associated with 2.8% (2.5, 3.1%) poorer balance time (Table 2, Figure 4). There was no evidence of an interaction with age for either height (*p* = 0.1) or BMI (*p* = 0.6) suggesting that the association stayed constant over time (Table 3).

**Table 3:** Summary of tests of non-linearity, sex interactions and age interactions of all covariates with balance performance.

	**Description of how variable is modeled**	**Sex interaction *p*-value**	**Age interaction effect on size of association**
Sex (female)	n/a^a^	n/a	↓ with age
**Socioeconomic indicators**		
Paternal occupational class	Continuously	0.9	Effect ↓ with age
Maternal education	Continuously	0.7	Effect ↓ with age
Education	Continuously	0.5	Effect ↓ with age
Own occupational class	Continuously	0.4	Constant with age
**Anthropometry**			
Height	Quadratic term	0.9	Constant with age
BMI	Linear term only	0.1	Constant with age
**Health behaviors**			
Leisure time physical activity	Categorically	0.7	Effect ↓ with age
Smoking	Continuously	0.1	Constant with age
**Current health status**			
History of diabetes	n/a^a^	0.5	Constant with age
History of cardiovascular events	n/a^a^	0.2	Constant with age
Respiratory symptoms	n/a^a^	0.6	Constant with age
Knee pain	n/a^a^	0.8	Effect ↑ with age
Symptoms of anxiety & depression	Linear term only	0.4	Effect ↑ with age
**Other**			
Verbal memory	Linear term only	0.2	Effect ↓ with age

a *Unable to test non-linearity in dichotomous indicators*.

### Health Behaviors and Balance

**Figure 5:**
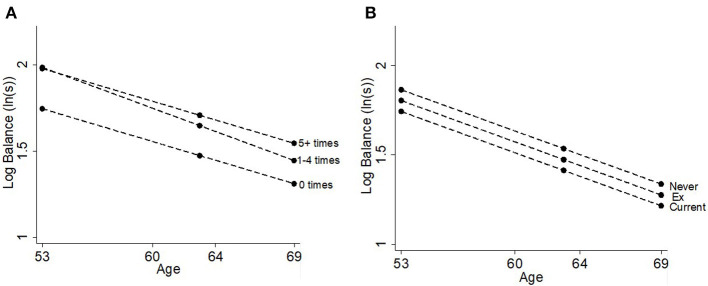
Differences in balance time at ages 53, 60–64, and 69 years by **(A)** leisure time physical activity and **(B)** smoking status.

Those who participated in leisure time physical activity 1–4 times [23.9% (17.3, 30.5%)] or 5+ times [23.3% (18.0, 28.7%)] per month had better balance times than those who did not participate at age 53 (Table 2, Figure 5A). There was no difference in balance between those who participated in leisure time physical activity 1–4 times/month and those who participated 5+ times/month. There was evidence that the association got smaller with age, as shown by the age-interaction for those who participated 1–4 times/month. Individuals who had a past history of smoking or who were current smokers had worse balance ability than those who had never smoked [6.1% (3.3, 8.9%); Table 2, Figure 5B]; there was no evidence that this association changed with age.

### Current Health Status and Balance

**Figure 6:**
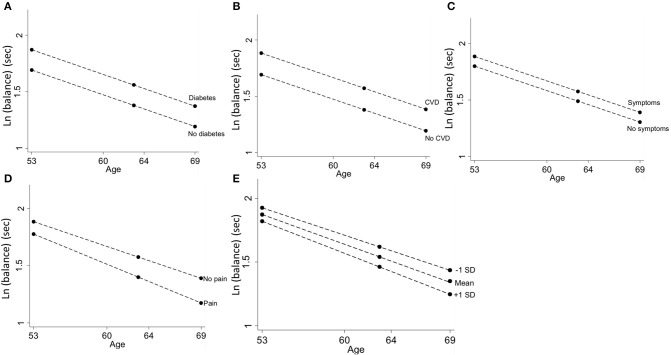
Differences in balance time at ages 53, 60–64, and 69 years by **(A)** diabetes, **(B)** CVD events, **(C)** respiratory symptoms, **(D)** knee pain, and **(E)** symptoms of depression and anxiety.

Individuals who had a history of diabetes, a history of CVD events or current respiratory symptoms had worse balance performance; these associations remained constant with age (Table 2, Figures 6A–C). Those who reported knee pain had 10.8% (4.6, 17.0%) lower balance times at age 53; this association got larger with age [0.7% per year (0.1, 1.2)] such that those with knee pain at age 69 had 21.6% (15.4, 27.8%) poorer balance than those with no knee pain (Table 2, Figure 6D). A one standard deviation increase in depression and anxiety symptoms on the GHQ-28 questionnaire was associated with a 5.2% (2.8, 7.7%, Table 2, Figure 6E) decrease in balance times. This association also increased with age by 0.3% per year (0.04, 0.5%); by age 69, 1 SD increase in GHQ-28 score was associated with a 9.5% (7.0, 11.9%) decrease in balance time.

### Cognitive Ability and Balance

**Figure 7:**
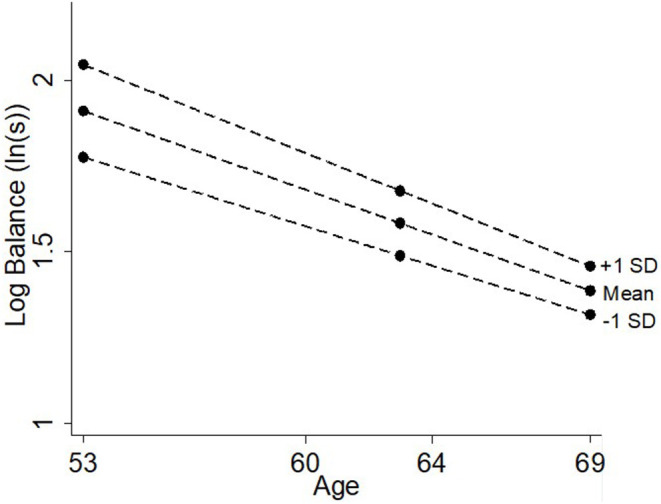
Differences in balance time at ages 53, 60–64, and 69 years by verbal memory.

One standard deviation increase in verbal memory was associated with a 13.4% (11.0, 15.9%; Table 2, Figure 7) increase in balance time at age 53. This association got smaller with age (0.5% per year, (0.3, 0.7%), but remained associated with balance at age 69 [5.1%, (2.6, 7.5%)].

### Combined Model of All Covariates and Their Association With Balance

Table 4 provides the estimates for the combined model of all covariates and the relevant age interaction terms. Notably, being female, having higher BMI, lower maternal education, lower educational attainment, lower own occupational class, not participating in leisure time physical activity, reporting a history of CVD events, higher levels of anxiety and depression and lower verbal memory remained associated with lower balance time. Nearly all age interaction terms weakened and were no longer statistically significant (at the 5% level) in this model, although there remained evidence that the associations with sex and verbal memory decreased with age.

**Table 4:** Combined model of all risk factors and all significant age interactions from individual models additionally adjusting for death and attrition, *n* = 2,465 (obs = 5,150).

	**Mean difference in % balance time at age 53 (intercept)**		Age (years)*risk factor interaction	
**Risk factors^a^**	**Coefficient (%) (95% CI)**	***p-value***	**Coefficient (%) (95% CI)**	***p-value***
Sex (female)	−21.7 (−28.7, −14.7)	<0.001	0.9 (0.4, 1.3)	<0.001
Paternal occupational class^b^ (per 1 level change)	−2.7 (−6.8, 1.5)	0.21	0.3 (−0.1, 0.7)	0.11
Maternal education^c^ (per 1 level change)	−3.9 (−6.7, −1.0)	<0.01	0.1 (−0.2, 0.3)	0.51
Education at age 26^d^ (per 1 level change)	−3.8 (−6.2, −1.3)	<0.005	−0.2 (−0.4, 0.02)	0.08
Own occupational class^b^ (per 1 level change)	−4.9 (−8.2, −1.7)	<0.005	–	–
Height (m)				
linear term	5.0 (−1.6, 11.6)	0.13	–	–
quadratic term	−0.02 (−0.04, 0.004)	0.12		
BMI (per kg/m^2^)	−2.1 (−2.5, −1.7)	<0.001	–	–
Leisure time physical activity^e^				
1–4 times/week	9.1 (1.9, 16.3)	<0.001	0.1 (−0.6, 0.8)	0.28
5+ times/week	5.8 (−0.2, 11.8)		0.3 (−0.3, 0.9)	
Smoking^f^ (per 1 level change)	1.4 (−1.6, 4.4)	0.36	–	–
Diabetes^g^	−6.9 (−14.1, 0.5)	0.07	–	–
CVD events^g^	−7.1 (−14.0, −0.3)	0.04	–	–
Respiratory symptoms^g^	−2.5 (−7.0, 2.0)	0.28	–	–
Knee pain^g^	−4.5 (−11.2, 2.1)	0.18	−0.2 (−0.8, 0.4)	0.55
Symptoms of depression/anxiety^h^ (per 1 SD)	−3.1 (−5.8, −0.4)	0.02	−0.1 (−0.4, 0.2)	0.46
Verbal memory^h^ (per 1SD)	5.9 (2.8, 8.9)	<0.001	−0.4 (−0.7, −0.1)	<0.01

a all models adjusted for sex as no evidence of sex interactions (see Table 3).

b *ref: I Professional or II Intermediate*.

c *ref: Secondary and further education*.

d *ref: Degree or higher*.

e *ref: none in last 4 weeks*.

f *ref: current smoker*.

g *ref: individuals with no health condition*.

h *SD estimates at each age are provided in Table 1*.

## Discussion

### Main Findings

We quantified associations between a range of risk factors across life and balance performance at ages 53, 60–64, and 69. Individuals with better balance were more likely to be male, have higher SEP in both childhood and adulthood, be taller, have lower BMI, partake in leisure time physical activity and were less likely to smoke. Individuals with better balance were also more likely to be healthier (no history of diabetes or CVD, not currently experiencing respiratory symptoms or knee pain), less likely to be experiencing symptoms of depression and anxiety, and more likely to have higher verbal memory. In a combined model, the majority of risk factors remained independently associated with balance, indicating that the factors across life that are associated with one-legged balance performance are multifaceted and complex.

Sex differences in balance performance were not explained by adjustment for other risk factors. Furthermore, there was no evidence to suggest that the associations between these risk factors and balance differed by sex, although several associations did change with age. Associations of balance performance with sex, socioeconomic indicators, physical activity and verbal memory became smaller with increasing age, while associations with anthropometric indicators, smoking, and physical health status stayed constant. Two associations became larger with age; associations of both knee pain and symptoms of anxiety and depression with balance doubled from age 53–69.

### Comparison With Other Studies and Explanation of Findings

#### Socioeconomic Indicators

A systematic review and meta-analysis of over 22 000 individuals from 11 separate studies reported that lower childhood SEP (as indicated by parental occupation and education) was associated with inability to balance with eyes open for ≥5 s (Birnie et al., 2011b); adjustment for adult SEP fully attenuated the effect of childhood SEP (paternal occupation used if available). However, maternal education and both indicators of adulthood SEP remained independently associated with balance time in our fully-adjusted model. In addition to differing operationalisations of one-legged balance performance (continuous vs. binary; eyes closed vs. eyes open), a possible explanation for these differing results is that 9 of the 11 studies included in the meta-analysis relied upon retrospective reports of childhood SEP (Birnie et al., 2011a). A strength of NSHD is that data on SEP and other risk factors were prospectively ascertained and so not prone to recall bias. As previously shown in relation to cognitive outcomes (Kaplan et al., 2001; Guralnik et al., 2006), paternal occupational class and maternal education may have distinctive associations with balance performance; further exploration of these differences are required.

While childhood SEP is hypothesized to be associated with balance ability via a complex pathway of health behaviors, education, adult SEP, cognitive ability and/or health conditions, the association remained when these factors were included in the model. Thus, childhood may also represent a sensitive period of development for balance ability, as previously hypothesized when testing associations of childhood cognition and midlife balance performance in NSHD (Blodgett et al., 2020). Adult SEP may also be associated with balance through a pathway of current physical and cognitive health or health behaviors. That both childhood and adult SEP indicators remained independently associated with balance suggests that accumulation of low SEP across the life course may be a greater risk factor than low SEP at any one particular life stage.

Notably, the relationship between most SEP indicators and balance time weakened with increasing age. This suggests that SEP may be more strongly associated with balance in midlife than at older ages when substantial age-related decline begins and chronic diseases manifest. Nevertheless, the association between the most recent measure of SEP (occupational class at age 53) and balance did not change with age.

#### Anthropometric Indicators

Higher body mass may influence the stability of an individual and the motor mechanisms involved in the balance process. For example, individuals with higher BMI often require more movement in order to maintain their balance, thus frequently demonstrate high levels of postural sway and reduced balance performance (D'Andréa Greve et al., 2013; Hita-Contreras et al., 2013). Studies have suggested that body stability is inversely related to the height of the center of gravity (D'Andréa Greve et al., 2013) and that shorter individuals are better able to maintain their balance. However, we found that taller individuals had better balance than shorter individuals though this effect appeared to plateau above a certain height.

Previous evidence has suggested that sex differences in balance performance disappear when scores are normalized to height (Maki et al., 1990; Hageman et al., 1995; Era et al., 2002; Bryant et al., 2005), while other studies have shown that anthropometric factors are major determinants of balance performance in women only (Kim et al., 2012). However, we found no sex differences in the association of either height or BMI with balance ability and adjustment for these measures did not explain sex differences (as seen in the combined model). Given that men have higher average strength and mobility compared to women (Miller et al., 1993; Sugimoto et al., 2014; Zunzunegui et al., 2015), further investigation into whether more detailed assessment of body composition (e.g., lean mass, fat mass) explains sex differences is warranted.

#### Health Behaviors

It is well-recognized that low levels of physical activity (de Rezende et al., 2014; Cooper et al., 2016; Olanrewaju et al., 2016; Schwingshackl et al., 2017) and current or past smoking (Cooper et al., 2016; Daskalopoulou et al., 2018) have negative consequences for an individual's physical capability, including their balance performance. Some studies have shown increasing levels of physical activity are associated with better balance (Powell et al., 2011; Cooper et al., 2015), while others have shown that there is no difference in health benefit between moderately active and maximally active groups (Cooper et al., 2016). In this study, participation in leisure time physical activity was associated with better balance performance. Although there was little additional benefit for balance ability beyond 1–4 times per month at age 53, a graded association between increasing levels of physical activity and balance performance emerged by age 69 (see Figure 5A).

Individuals who currently smoked had worse balance performance compared with those who were ex-smokers; ex-smokers also had worse balance compared with those who had never smoked. Although not previously examined in balance ability, this is consistent with increasing severity of poor physical capability seen amongst categories of smoking history (North et al., 2015; Cooper et al., 2016), suggesting that quitting smoking may have a positive association with balance performance.

#### Current Health Status

The presence of each physical and mental health condition (diabetes, CVD, respiratory symptoms, knee pain, symptoms of anxiety, and depression) was associated with poorer balance performance. This is consistent with the literature on how current health impacts an individual's physical capability or functional decline (Welmer et al., 2012; Kuh et al., 2014; Ryan et al., 2015). Each health condition likely has a direct biological pathway impacting balance. For example, diabetes is related to both peripheral neuropathy (Greene et al., 1992) and age-related visual impairment (Lutty, 2013; Pelletier et al., 2016) while knee pain can have a direct impact on proprioception and musculoskeletal function (Sanchez-Ramirez et al., 2013). Individuals with a history of CVD events or respiratory symptoms often demonstrate shared pathophysiological features common in those with balance impairment including increased postural sway due to physical displacement of breathing (Jeong, 1991), decreased blood flow in specific functional areas (Abate et al., 2009) and decreased musculoskeletal capacity (Crisan et al., 2015). Finally, increased inflammatory markers that are common in arthritis, such as C-reactive protein or nitric oxide (Cepeda et al., 2016) are also more common in individuals with depression than those without. In addition to this inflammation pathway, individuals with depression also tend to restrict their physical activity, have reduced motivation to perform well and exhibit psychomotor impedance such as a slowing in musculoskeletal components (Bennabi et al., 2013); all of these factors can influence balance performance.

The associations of diabetes, CVD and respiratory symptoms with balance performance were constant. However, the associations of knee pain and symptoms of anxiety and depression with balance got stronger at older ages. The constant or increasing associations between health conditions and balance ability with age suggests that overall health becomes relatively more important for balance ability in later life; this could in part explain why the strength of associations with many other risk factors decreased with increasing age.

#### Cognitive Ability

As expected given previous findings in NSHD (Kuh et al., 2009a; Blodgett et al., 2020), higher verbal memory was associated with higher levels of balance performance. Cognitive processing of sensory and motor input is an important component of the balancing process (Li et al., 2018). Previous evidence in NSHD has shown that childhood cognitive ability is associated with adult balance performance; this is primarily via an adult cognition and education pathway that is independent of most of the other risk factors examined here (Blodgett et al., 2020). As suggested above, the decreasing strength of association with age suggests that cognitive ability becomes less important with age, as other factors in the aging process, in particular health conditions, become more important.

### Methodological Considerations

A major strength of this paper is the assessment of balance performance at three ages, which facilitated our novel investigation into whether associations between risk factors and balance ability change over 16 years, from mid to later life. A second strength is the comprehensive investigation, in separate and combined models, of the associations between 14 different factors across life and balance performance. These risk factors were all prospectively ascertained which increases reliability of response and limits recall bias. A third strength was the methods used to include those individuals who had missing balance scores for health reasons or because of death or loss to follow up between ages 53 and 69. These combined strengths provided novel evidence on how the associations between these risk factors and balance ability change with age. Finally, the age homogeneity of the sample eliminated any confounding by age that is common when examining physical capability in mid and later life (Seeman and Chen, 2002; Garber et al., 2010).

One potential limitation of our study is that we were unable to include participants in analyses if they had been lost to follow up before age 53 (i.e., the age at which balance was first assessed). Characteristics of study members who were lost to follow up before the first clinical assessment at age 53 were more likely to be male (Stafford et al., 2013), have lower childhood and adulthood occupational class (Stafford et al., 2013; Kuh et al., 2016), demonstrate unhealthy behaviors (smoking, physical inactivity) (Stafford et al., 2013), have lower verbal memory (Stafford et al., 2013) and have poorer overall health (Kuh et al., 2016). Participants who were followed up to age 53 but could not be included in analyses due to missing data on risk factors had similar characteristics to those lost to follow-up before age 53. Many of these characteristics (i.e., low SEP, unhealthy behaviors, lower cognition, and poorer health) were negatively associated with balance ability, thus it is hypothesized that those with lost to follow up before age 53 or with missing risk factor data may have had poorer balance. This likely resulted in an underestimation of the size and strength of associations.

Two further limitations are the assumptions of the model: that the change in balance over time is linear and that all individuals follow the same mean trajectory of decline. Individuals are likely to exhibit heterogonous aging trajectories as they demonstrate different patterns of change in balance performance with age (e.g., steeper decline, delayed decline, maintenance of balance ability). As there were only three time points for balance, it was not appropriate to test for non-linearity in balance trajectories. Identifying polynomial time terms can help identify the age at which decline begins or accelerates. Further research, with at least four measures of balance performance, should address these differences across time and between individuals. We also need to consider the possibility that other factors not considered in our analyses such as alcohol consumption and medication use may also be important and need to be considered in future research.

Although there were multiple comparisons, the risk of Type 1 error remains low as all of the primary associations in Table 2 were significant at *p* < 0.001 and an alpha of 0.05 was intentionally used for interaction terms to ensure a parsimonious model. Finally, one-legged balance ability measures a specific aspect of static balance that does not directly represent the dynamic balance relied upon in everyday situations (Owings et al., 2000; Mackey and Robinovitch, 2005; Bhatt et al., 2011). Further research should consider if associations between the risk factors identified in this study are consistent for tests of dynamic balance or for more sensitive measures of postural sway, as assessed with a force plate.

### Implications and Conclusions

We investigated 14 different factors across life that are associated with balance performance. These findings are important in considering appropriate interventions to minimize balance decline or when identifying high-risk individuals. That multiple risk factors were identified suggests that a multifactorial approach including behavioral, health and cognitive factors (amongst others) may have more benefit than a focus on a single risk factor. As several of these risk factors have different associations with balance at different ages, there may be benefit in targeting different factors at different ages. Knee pain and symptoms of depression and anxiety both appear to become more important with age and may represent important targets for intervention in midlife before their potential association with balance performance increases. While not all of the factors identified (e.g., socioeconomic, height, smoking history) may be easily modified, they are likely to have utility in helping to identify individuals at high risk of future balance difficulties who may require more support than others to maintain balance ability as they age.

In summary, this study identified many anthropometric, behavioral, socioeconomic, health and cognitive risk factors across life that are associated with balance ability. The majority of variables remained independently associated with one-legged balance performance, suggesting that the range of risk factors associated with poor balance ability is diverse and complex. This highlights the importance of considering both type (i.e., multifactorial approach) and timing (i.e., early, mid, and later life) of interventions that target balance performance in adulthood.

## Data Availability Statement

The datasets used in this study will not be made publicly available. Access to NSHD data adheres to strict confidentiality guidelines but these data are available to bonafide researchers upon request to the NSHD Data Sharing Committee via a standard application procedure. Further details can be found at http://www.nshd.mrc.ac.uk/data. doi: 10.5522/NSHD/Q101; doi: 10.5522/NSHD/Q102; doi: 10.5522/NSHD/Q103.

## Ethics Statement

At each wave of data collection, relevant ethical approval has been received. Ethical approval for the most recent data collection (2014–2015) was obtained from Queen Square Research Ethics Committee (13/LO/1073) and Scotland A Research Ethics Committee (14/SS/1009). All participants have provided written informed consent.

## Author Contributions

JB performed statistical analyses and wrote the first draft of the manuscript. All authors contributed to the conception and design of the study, to manuscript revision, read, and approved the submitted version.

### Conflict of Interest

The authors declare that the research was conducted in the absence of any commercial or financial relationships that could be construed as a potential conflict of interest.
